# Enteral administration of antiepileptic agents could have efficacy for prevention of post-traumatic seizures in severe traumatic brain injury

**DOI:** 10.1186/cc13671

**Published:** 2014-03-17

**Authors:** K Mocjiduki, H Suzuki, A Kawasaki, M Hotta, M Namiki, T Harada, M Takeda, A Yaguchi

**Affiliations:** 1 Tokyo Women's Medical University, Tokyo, Japan

## Introduction

Antiseizure prophylaxis is recommended for preventing only early post-traumatic seizures (PTS) in the guidelines for the management of severe traumatic brain injury (TBI) by the Brain Trauma Foundation. Phenytoin is recommended to reduce the incidence of early PTS prophylaxis. Early enteral nutrition has recently shown theoretical advantages for prevention of bacterial translocation to maintain normal turnover of gut mucosa and is commonly used for TBI patients. Our hypothesis is that the enteral administration of antiepileptic agents is also useful for early PTS.

## Methods

This retrospective observational study included all adult patients admitted to our tertiary academic medico-surgical ICU due to TBI from September 2011 to August 2012. Patients who have epilepsy as a past history were excluded. Clinical data were collected from electrical medical archives. The baseline characteristics collected were age, gender, diagnosis, antiepileptic agents, timing of start and adverse effects of those agents, and methods of administration.

## Results

Of 65 patients with TBI, 25 patients (18 men, seven women; mean age 56.7 ± 20.1) who were administered antiepileptic agents for PTS prophylaxis were studied. Fifteen cerebral contusions, 10 acute subdural hematomas, nine traumatic subarachnoid hemorrhages, two cerebral infarctions, two pneumocephalus and one traumatic intracerebral hemorrhage were shown in 25 patients. All patients were alive 28 days after the injury. Fourteen patients (56%) were intravenously administered (13 phenytoin and one phenobarbital), while 11 patients (44%) were administered with enteral feeding (four valproates, four carbamazepine and three zonisamides) as PTS prophylaxis. The average start day of PTS prophylaxis was 2.6 days after the injury by intravenous administration, and 2.2 days by enteral administration, respectively. Two patients with phenytoin showed hepatic dysfunction as an adverse effect and no patient showed early PTS both by intravenous and by enteral administrations.

## Conclusion

The present study has some limitations because it is a single-center retrospective analysis. However, enteral administration of antiepileptic agents could be useful for PTS prophylaxis. Considering cost, adverse effects and serum monitoring, there is a possibility of enteral administration of antiepileptic agents as an alternative to intravenous phenytoin.

